# Using Peptidomimetics and Constrained Peptides as Valuable Tools for Inhibiting Protein–Protein Interactions

**DOI:** 10.3390/molecules23040959

**Published:** 2018-04-19

**Authors:** Naomi S. Robertson, David R. Spring

**Affiliations:** Department of Chemistry, University of Cambridge, Lensfield Road, Cambridge CB2 1EW, UK; nsr32@cam.ac.uk

**Keywords:** protein–protein interactions, peptidomimetics, proteomimetics, macrocycles, stapled peptides

## Abstract

Protein–protein interactions (PPIs) are tremendously important for the function of many biological processes. However, because of the structure of many protein–protein interfaces (flat, featureless and relatively large), they have largely been overlooked as potential drug targets. In this review, we highlight the current tools used to study the molecular recognition of PPIs through the use of different peptidomimetics, from small molecules and scaffolds to peptides. Then, we focus on constrained peptides, and in particular, ways to constrain α-helices through stapling using both one- and two-component techniques.

## 1. Introduction

Protein–protein interactions (PPIs) are well-recognised as mediators of a plethora of processes in biological systems and are vitally important in the progression of many disease states [1,2,3]. There are estimates of between 130,000 to 650,000 relevant interactions in the human protein–protein interactome [4,5,6]. Many of these interactions are underexplored and so represent an emerging area for drug discovery. Through the study of PPIs, targets that were previously overlooked have now come to light as significant targets of interest.

This review will focus on the methods used to target PPIs, from small molecules, peptidomimetics and peptides and, in particular, constrained peptide macrocycles and stapled peptides.

## 2. General Structure of PPIs

The development of new therapeutics which target PPIs is inherently challenging for medicinal chemistry and chemical biology [7]. Most interactions are dynamic and occur over a relatively large protein contact surface area (1500 to 3000 Å^2^) [8,9], much larger than the average contact area needed for small molecule binding, which is thought to be approximately 300 to 1000 Å^2^ [10]. Additionally, PPIs have generally been thought of as undruggable because many protein–protein interfaces lack the obvious pockets for binding small molecules [11]. Constitutive PPIs tend to be predominantly driven by hydrophobic effects [1], while transient PPIs consist of more polar residues [12]. Proteins which have transient PPIs can often have more than one binding partner from a much larger protein family. A well-known example comes from the Bcl-2 family of proteins, where prosurvival members of the family interact with the proapoptotic BH3 (Bcl-2-homology-3)-only members [13].

While a PPI is usually large in size, not all residues contribute to binding equally and, in fact, only a small number of crucial amino acid residues within the PPI are important in delivering the vast majority of the binding affinity and specificity [14,15,16,17,18]. These regions have been termed ‘hot spots’. Many of these have been elucidated through systematic alanine scanning mutagenesis, where a hot spot residue is established if, when that residue is mutated to an alanine, the binding energy difference is more than 2 kcal/mol [15]. Hot spots were found to be rich in residues such as arginine, tryptophan and tyrosine.

Although PPI hot spots are presented on the protein surface, these residues are generally not contiguous within the protein sequence as a result of protein folding. Many hot spots are associated with protein secondary structure motifs such as the α-helix, β-sheet and β-turn. Of these secondary structures, the α-helix has captured the interest of researchers. This is because α-helices comprise approximately 60% of all secondary structures in protein complexes [18,19]. Additionally, α-helices have been shown to mediate a large number of key therapeutically relevant PPI interfaces, of which 60% bind to one face of the helix [19]. At the protein–protein interface, α-helices tend to bind into the groove of their binding partner, and as a result, helix mimetics have been of great interest. A number of methods exist which aid in the discovery of new PPIs and facilitate the discovery of molecules to bind PPIs, including high throughput screening [20], phage display [21], crosslinking [22], computational studies [23] and structural based design, to name a few.

## 3. Methods for Targeting PPIs

### 3.1. Small Molecules and Peptidomimetics

Traditionally, peptidomimetics have been subdivided into three types [24,25,26]. Type I mimetics are short peptides which mimic the secondary structure landscape of the parent peptide, with minor alterations to the sequence. Type II mimetics are non-peptidic functional molecules based on a scaffold that does not mimic the peptide secondary structure. Type III mimetics are also non-peptidic molecules and these match the spatial topology of key interaction motifs of the parent peptide. More recently, these categories have been further improved by Pelay–Gimeno et al. into four different classes: Classes A–D, where Class A mimetics are most similar to the parent peptide, while Class D mimetics show the least similarities [26]. Class A mimetics, like Type I mimetics, are peptides with minimal alterations to the peptide side chains and backbone. Class B mimetics, while still peptidic in nature, include much more dramatic backbone and side chain alterations (e.g., peptoids, β-peptides and α/β-mixed peptides). Class C mimetics are similar to Type II mimetics and involve a scaffold, from which substituents that are analogous to the peptide side chains are projected. Finally, Class D mimetics are those that mimic the mode of action of a peptide without a direct link to the peptide side chains.

**Figure 1:**
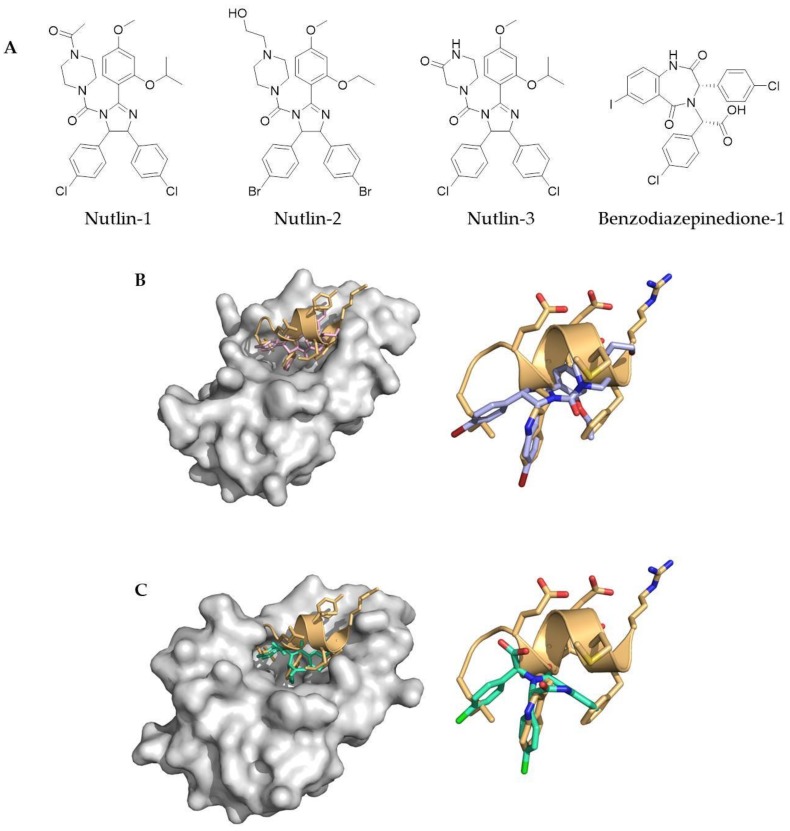
(**A**) Classic small molecule inhibitors of protein–protein interactions (PPIs) include the Nutlin family of small molecules (Nutlin-1, Nutlin-2 and Nutlin-3) and benzodiazepinediones; (**B**) crystal structure of Nutlin-2 bound in the p53 binding pocket of MDM2 (PDB 1RV1), overlaid with a p53 helix (PDB 1T4F) (left) and showing the overlap between Nutlin-2 and the side chain residues of p53; (**C**) crystal structure of benzodiazepinedione-1 bound in the p53 binding pocket of MDM2 (PDB 1T4E), overlaid with a p53 helix (PDB 1T4F) (left) and showing the overlap between benzodiazepinedione-1 and the side chain residues of p53.

Classical medicinal chemistry uses small molecule drugs which bind either into the active site of a protein, or at an allosteric position. However, as alluded to earlier, PPIs tend to be large and challenging to target with small molecules. Nevertheless, a number of small molecule PPI inhibitors have successfully been developed; including ABT-737 and ABT-236, which both inhibit the Bcl-x_L_/Bak PPI (Abott Laboratories) [27,28], along with the Nutlin family of small molecule compounds (Hoffmann-La Roche) and the benzodiazapinediones (Johnson & Johnson Pharmaceuticals), which inhibit the p53/mDM2 PPI [29,30] (Figure 1). These are all examples of Class D mimetics.

**Figure 2:**
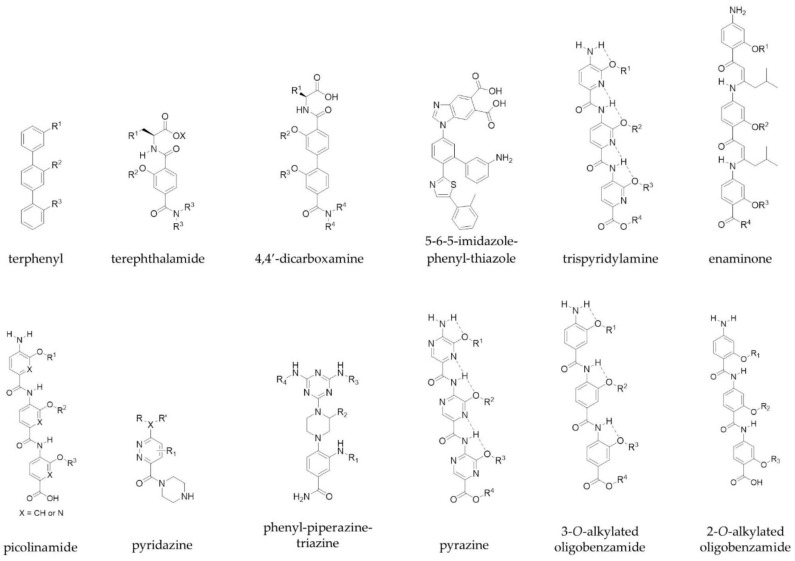
Examples of the proteomimetic scaffolds used to target PPIs. R groups mimic peptide side chains of hot spot residues.

Class C mimetics include scaffolds such as terphenyls, which were established by Hamilton and colleagues, and mimic one face of an α-helix in order to target PPIs [31]. They developed a series of trisubstituted 3,2′,2″-terphenyl compounds, the aryl cores of which adopt a staggered conformation (dihedral angles, 59.1° and 120.7°) and thus mimic the *i*, *i+3*, *i+4* and *i+7* residues of a helix through the *ortho* positions of the scaffold [31]. In addition, other scaffolds such as terephthalamides [32], 4,4′-dicarboxamines [33], 5-6-5-imidazole-phenyl-thiazoles [34], trispyridylamines [35] and enaminones [36] have been developed as extended α-helix mimetics by the Hamilton group. Other groups have reported their own scaffolds to mimic amino acid side chains on α-helices. These scaffolds include picolinamides [37], pyridazines [38,39], phenyl-piperazine-triazines [40], pyrazines [41], 3-*O*-alkylated oligobenzamides [42] and 2-*O*-alkylated oligobenzamides [43] (Figure 2), and mimic one face of the helix. Notably, the Wilson group were the first to describe a solid-phase synthesis for an α-helix mimetic with *N*-alkylated oligobenzamides, which act as inhibitors of p53/hDM2 [44,45]. The same group were also able to show orthogonal functionalisation of these non-peptidic helix mimetics through a copper-mediated ‘click’ chemistry approach [46].

**Figure 3:**
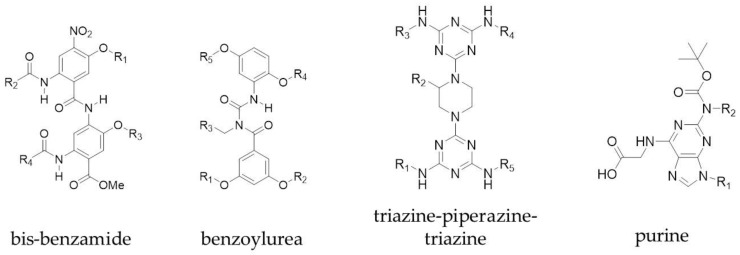
Examples of multi-faced proteomimetic scaffolds used to target PPIs. R groups mimic peptide side chains of hot spot residues.

Peptidomimetics that mimic more than one face of an α-helix have also been explored. Both the Ahn and Hamilton groups converted a single faced bis-benzamide scaffold into a dual faced helix peptidomimetic (Figure 3) [47,48]. Additionally, an amphiphilic α-helix mimetic based on a benzoylurea scaffold has been reported by Thompson and Hamilton [49], while Lee et al. developed two-face amphipathic α-helix mimetics based on a triazine-piperazine-triazine scaffold [50] (Figure 3). The triazine-piperazine-triazine peptidomimetics showed improved binding affinity to Mcl-1 in a fluorescence anisotropy competition assay compared to a fluorescein labelled BH3 peptide. More drug-like proteomimetics based on a purine scaffold have also been reported by Lanning et al. (Figure 3) [51]. A number of other non-peptidic scaffolds have also been reported which mimic more than one face of the helix and a review of these has been published by Lanning and Fletcher [52].

### 3.2. Peptides to Target PPIs

Short peptide sequences are associated with a number of problems. They often lack the ability to fold into their bioactive conformation because of an entropic penalty for folding and they are susceptible to faster rates of degradation due to proteolysis. Overcoming these difficulties frequently involves one or more of the following: incorporation of non-native amino acids [53], D-amino acids [54,55,56,57], and β-amino acids [58]; retro-inverso peptides [59]; changing the backbone (through *N*-methylation [60], or the introduction of amide bond isosteres [61]); and cyclising strategies [62,63,64,65]. Non-native amino acids confer stability as a result of the proteolytic machinery in the cell being ill equipped to deal with unnatural amino acids [66]. Unnatural amino acids such as α-aminobutyric acid (Aib or α-methylalanine) can promote secondary structure formation (3_10_-helix), while other α,α-disubstituted amino acids, such as α-pentenylalanine, can help form an α-helix, owing to the Thorpe–Ingold effect [67]. Incorporation of D-amino acids also inverses the stereochemistry at the α-carbon of the peptide backbone, and a fully retro-inverso peptide not only inverts the chirality of the peptide but also reverses its sequence. This in turn gives a peptide with the side chains projected in the correct orientation [68]. Backbone modifications such as *N*-methylation can alter the *cis–trans* configuration of the amide bond, affecting the conformational freedom of adjacent amino acids [26]. It also alters the hydrogen-bond pattern of the peptide through reducing the number of hydrogen bond donors. Other backbone modifications, such as the use of foldamers, β-peptides and peptoids can additionally infer both proteolytic and metabolic stability [69,70]. Cyclic peptides have improved metabolic stability compared with their linear counterparts and cyclisation introduces conformational constraints that can reduce the flexibility of the peptide, allowing for a reduced entropic cost upon binding, thus increasing binding affinity [66,71]. A few of the above peptide modifications will be discussed further in the following Sections.

### 3.3. Macrocyclic Peptides

Macrocyclic scaffolds are found in many natural products such as cyclosporine A, sunflower trypsin inhibitor (STF-1) and Rhesus θ defensin 1 (RTD-1); as a result, synthetic macrocycles have been widely investigated in the development of novel therapeutics and chemical probes [26,72,73].

**Figure 4:**
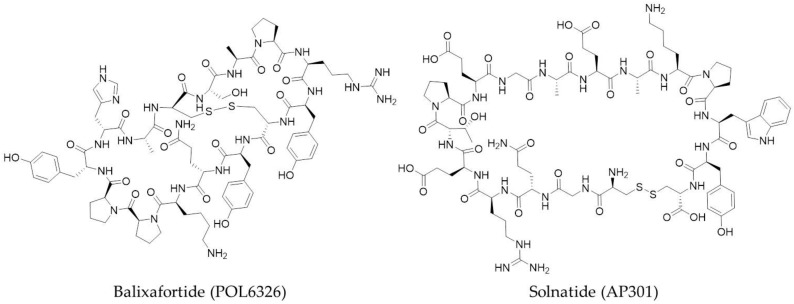
Two examples of macrocyclic peptides (Class A PPI inhibitors) which are currently undergoing clinical trial studies as PPI inhibitors: Balixafortide (POL6326) from Polyphor and Solnatide (AP301) from Apeptico.

A number of pharmaceutical companies have macrocyclic peptides currently undergoing clinical trials for a range of targets. Examples include Polyphor, who have two drugs in clinical trials, Balixafortide (POL6326) (Figure 4) and POL6014; Bicycle Therapeutics, with bicyclic peptide BT1718; Apeptico, with Solnatide (AP301) (Figure 4); Ra Pharmaceuticals with RA101495; and Aileron Therapeutics, with two drugs, ALRN-5281 and ALRN-6924, also in clinical trials.

Balixafortide is a bicyclic peptide that is a potent and selective agonist of the chemokine receptor CXCR4. In combination with eribulin (Halaven^®^), balixafortide has successfully completed a Phase 1 study for the treatment of advanced metastatic breast cancer and other oncology indications [74]. The structure of POL6014 has not yet been released, but the successful conclusion of its Phase 1 clinical study has recently been announced for the treatment of cystic fibrosis, non-cystic fibrosis bronchiectasis and alpha 1 antitrypsin deficiency [75].

BT1718, developed by Bicycle Therapeutics, has recently entered a Phase 1/2a study in solid tumours. It is a constrained bicyclic peptide which binds to membrane type 1-matrix metalloprotease (MT1-MMP; MMP14) and is a first-in-class bicyclic drug conjugate [76]. Developed by Apeptico, Solnatide has continued into Phase 2 clinical trials after a successful first-in-man study to assess the safety of the orally inhaled aerosol [77]. It is a cyclised peptide of the lectin-like domain on human TNF-α and an activator of ENaC-mediated Na^+^ uptake for the treatment of pulmonary permeability oedema in acute respiratory distress syndrome (ARDS). Now in Phase 2 clinical trials for paroxysmal nocturnal hemoglobinuria, RA101495, developed by Ra Pharmaceuticals, is a potent cyclic peptide inhibitor of complement component 5 (C5) [78].

A subset of macrocycles is concerned with conformationally constraining α-helices. This practice has been termed peptide stapling. Stapled peptides have recently come of age, with two compounds developed by Aileron Therapeutics in clinical trials.

## 4. Stapled Peptides

In general, the entropy of folding is the limiting factor for short isolated peptide sequences to fold into their bioactive conformation. Consequently, the stabilisation of these peptides has been studied extensively. However, additional factors such as the entropy contribution made by the desolvation of water molecules has also been shown to be important [79]. Synthetically constraining a peptide can reduce the entropy of folding, however, it may also restrict the peptide from folding into the correct conformation for binding [79,80]. Even so, for α-helical peptides, ‘stapling’ has come to the forefront as a viable method of introducing constraints, where the side chains of two residues in the peptide are covalently linked to form a macrocycle. Peptide stapling can increase α-helical character, protease stability, binding affinity and promote cell penetration when compared to their unmodified counterparts [81].

There are an abundance of different techniques that have been developed for peptide stapling. The different methods for peptide stapling can be divided into two subsets: one-component stapling, where the side chains of two amino acids are directly linked; and two-component stapling, where the sides chains of two amino acids are connected through a linker.

### 4.1. One-Component Stapling

One-component stapling methods have been developed to allow the use of both natural (e.g., lysine, glutamic acid etc.) and non-natural (e.g., alkenyl and azido amino acids) amino acids as a stapling anchor. The earliest form of stapling did not involve covalent linkage, but instead used salt bridges between complementarily charged residues, particularly lysine and glutamic acid residues [82]. However, with these peptides, the environment the peptide occupies plays a role in inducing the peptide conformation, thus care must be taken to control both pH and salt concentrations [83]. Since salt bridges, a number of other one-component stapling techniques have been reported, including lactamisation [84,85,86], triazoles [87] and all-hydrocarbon stapling [79,88,89,90,91,92,93], to name a few.

Rosenblatt and co-workers introduced the idea of lactam staples, where the proteinogenic amino acids lysine and aspartic acid at *i* and *i+4* positions on a short sequence of parathyroid-hormone-related protein (PTHrP) were cyclised [84]. Lactamisation has since been systematically studied by Fairlie and co-workers on simple pentapeptides, where they showed that a single lactam bridge could effectively stabilise short α-helical peptides, while also being aqueous stable [94]. Additionally, they demonstrated the use of consecutive lactam bridges to constrain α-helices [95]. With these data in hand, Fairlie and co-workers applied this stapling strategy to a number of biologically relevant targets, including doubly lactam-stapled peptide analogues of hormone nociception, which induced greater levels of ERK phosphorylation in cells and thermal analgesia in mice [96].

‘Click chemistry’ was introduced by Sharpless in 2001 [97], and from the initial use of the Huisgen 1,3-dipolar cycloaddition on peptides by Meldal and co-workers, the copper catalysed azide alkyne click (CuAAC) reaction has catapulted into everyday use [98]. A notable one-component example of CuAAC comes from Kawamoto et al. who carried out a systematic study on the effect of linker length, position of the staple within the peptide and the effect of stereoisomers for the residue used for stapling [87]. They found that for an *i,i+4* triazole stapled peptide, the best binding affinity peptide for targeting the β-catenin/B-cell CLL/lymphoma 9 (BCL9) PPI, was where the residues used for stapling were l-amino acids. This peptide also gave the greatest increase in helicity.

An interesting example of one-component stapling comes from Yamagishi et al. who reported a cyclisation strategy which involved the oxidative coupling of 5-hydroxyindole and benzylamine, which were introduced into the peptide through non-proteinogenic amino acids [99]. The cyclisation of a non-fluorescent peptide occurred rapidly at room temperature after the addition of K_3_Fe(CN)_6_ to generate a conjugated, heterocyclic linker structure which conferred fluorescence. Here, the linker not only acts as a peptide constraint but also as a fluorescent probe.

The current ‘gold standard’ for one-component stapling is the all-hydrocarbon staple, pioneered by Miller, Blackwell and Grubbs in the late 90s [100,101,102]. The field has since erupted through the work of the Verdine and Walensky groups [81,88,92,103,104,105]. All-hydrocarbon stapling relies on using the ruthenium catalysed ring closing metathesis reaction to form the staple macrocycle. Unnatural alkenyl amino acids which can be difficult to synthesise are also required for this ring closing strategy [106]. These are likely to be α,α-disubstituted amino acids, though some examples have been reported using monosubstituted alkenyl amino acids [107,108]. The most widely used all-hydrocarbon staple constrains α-helical peptides across a single turn (*i,i+4*). However, *i,i+3* [90,109] and *i,i+7* [89] have also been reported, as well as doubly stapled [110] and stitched stapled peptides [111].

The importance of all-hydrocarbon stapling has recently been highlighted as the result of ALRN-5281, a long-acting growth-hormone-releasing hormone agonist for the treatment of orphan endocrine diseases, successfully completing its Phase I clinical trial [112]. Additionally, Aileron Therapeutics also have another stapled peptide drug (ALRN-6924) in Phase I clinical trials for solid tumours and in Phase II trials for lymphoma and peripheral T-cell lymphoma [113,114]. ALRN-6924 is a stapled peptide designed to disrupt integration between the p53 tumour suppression protein and inhibition by murine double minute 2 (MDM2) and murine double minute X (MDMX). The results for these studies should be released in July 2018.

### 4.2. Two-Component Stapling

**Figure 5:**
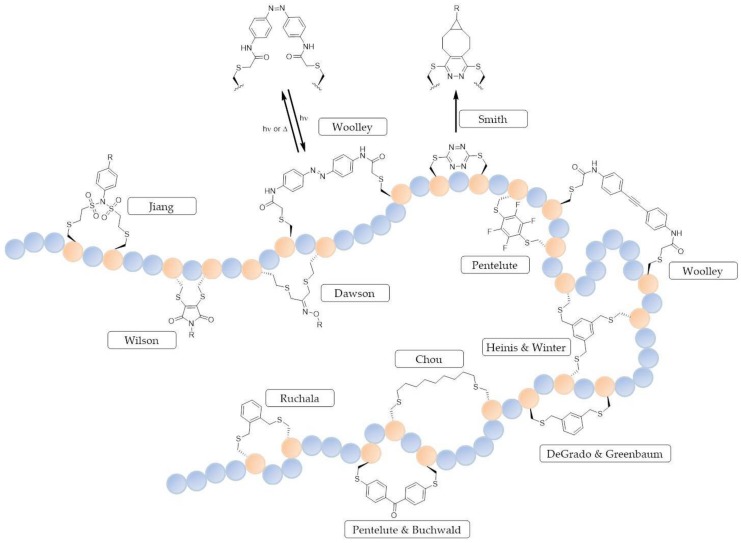
Some of the reported two-component cysteine cyclisation strategies, where R = different functional tags and handles.

A number of two-component stapling techniques have been reported including photocontrollable macrocycles [115,116,117,118] and the use of bridging motifs like alkyl chains [119,120], aromatics [120,121,122,123,124], perfluoroaryl [125] and tetrazine [126]. These examples have been carried out using the natural amino acid cysteine as an attachment point (Figure 5), but other examples have also been reported for two-component stapling using lysine and tryptophan residues [127,128] and non-native amino acids containing alkyne functionalities [129,130,131,132,133]. Cysteine has been exploited for two-component stapling partly due to the high nucleophilicity of the sulfhydryl group which can readily undergo alkylation with suitable electrophiles such as α-halocarbonyls and Michael acceptors [134]. An excellent review on stapling using cysteine crosslinking has been published by Fairlie and de Araujo [135].

The ability to control the activity of a peptide through an external stimulus, such as light, has led to the development of photocontrollable macrocycles, introduced by the Woolley group [115,116,117]. Photosensitive azobenzene linkers were installed to give macrocycles across the *i,i+4*, *i,i+7* or *i,i+11* positions on the peptide sequence. The *trans*-to-*cis* isomerisation of the azobenzene linker produced a conformational change in the peptide to give either an α-helix or random coil. Furthermore, the Woolley group employed a structurally rigid ethylene-based linker to stabilise across *i,i+11* (three turns) of an α-helix [136]. For a comprehensive review on the azobenzene photocontrol of peptides and proteins, see Mart and Allemann [137].

DeGrado and Greenbaum showed that an aromatic linker such as dibromo-*m*-xylene reacts with the sulfhydryl cysteine side chain in a simple one-pot reaction, both in solution and on solid support to constrain an α-helical peptide and increase its helicity [120]. This strategy was applied to the stapling of calpain probes to mimic a natural PPI of cysteine proteases with good potency and selectivity. Another *S*-alkylation approach comes from Micewicz et al., who synthesised analogues of a potent dual-specific antagonist of p53–MDM2/MDMX interactions, PMI-N8A [124]. They showed the stapled peptide to be cell-permeable and displayed potent anticancer activity at a low dose (0.3 mg/kg) against a human colorectal cancer cell line.

Bicyclic peptides linked through cysteine residues are of great interest, especially with the clinical trial of a bicyclic peptides developed by Bicycle Therapeutics. The initial work on this strategy was carried out by Heinis and Winter, where a series of peptides with three reactive cysteine residues, spaced by six amino acids, were synthesised and fused to the phage gene-3-protein. The peptides were then conjugated with 1,3,5-tris(bromomethyl)benzene under mild aqueous conditions to generate a series of bicyclic peptides covalently attached to the mesitylene core [138]. This approach has been used to synthesise a potent and selective inhibitor of human urokinase-type palasminogen activator (uPA) [139]. Apart from tris(bromomethyl)benzene, a number of other small molecule linkers with thiol-reactive groups have been reported [140].

Dichloroacetone (DCA) was incorporated as a staple linkage by Assem et al. to enhance helical secondary structure [141]. Following stapling, the ketone moiety of the linker was then modified with a diverse range of molecular tags (fluorophore, CPP etc.) through oxime ligation. Additionally, a second macrocycle was formed through the cyclisation of an *N*-terminal aminooxy group with the acetone linker.

A rapid and reversible two-component stapling methodology, using cysteine or homocysteine as the peptide anchoring amino acids, has recently been reported by Grison et al. [142]. The staple is formed through employment of a dibromomaleimide, which can be further functionalized by ‘click’ chemistry. This reversible approach has been hypothesised to allow for the delivery of a peptide-based reagent into the cell, where the constraint can be removed, and thus the peptide is less readily transported out of the cell [142].

Tetrazine staples were installed as a constraint by Brown and co-workers through the use of a dichlorotetrazine linker reacting with two cysteine residues between two and 27 residues apart [126]. Additionally, they functionalized the linker following macrocyclisation with the use of an octyne-derived fluorescein probe through an inverse-electron demand Diels-Alder reaction. Another S_N_Ar linker has been reported by the Pentelute group using a perfluoroaryl linker, which has the benefit that the peptide is adorned with NMR-detectable ^19^F atoms which is a useful analytical tool [125]. In addition, the Pentelute and Buchwald groups have expanded on the S_N_Ar bioconjugation methodology with the use of a bis-palladium complexed benzophenone [122].

Photoinduced coupling of cysteines and alkenes, also known as thiol-ene coupling, has recently been investigated by Wang and Chuo using an α,ω-diene, while using an initiator with irradiation at 365 nm to install a thioether linkage across *i,i+4*, *i,i+7* and *i,i+8* positions [119]. More recently, *N*-phenyl-divinylsulfonamides have been investigated for two-component cysteine stapling by Li et al., where they treated oxytocin with *p*-CH_3_O-*N*-phenyldivinylsulfonamide linkers functionalized with different handles [143].

An intramolecular tryptophan condensation approach for peptide stapling was recently reported by Hui et al. using an aldehyde under mild acidic conditions [128]. The tryptophan residues were linked at the C2 position of the indole to give a variety of *i,i+n* (where *n* = 1,2,3 or 4) stapled peptides.

**Figure 6:**
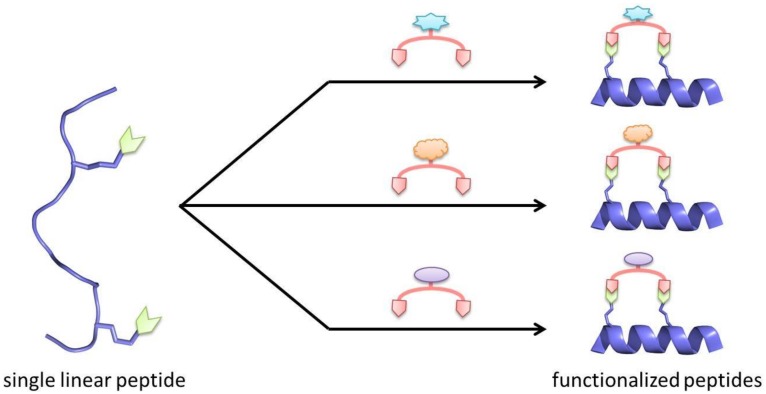
From a single synthesised linear peptide, a large library of functionalized stapled peptides can be made through the use of a variety of linkers, which can include fluorophores, cell penetrating peptides and handles for pull down assays.

As alluded to in some of the examples above, unlike one-component stapling techniques, two-component stapling also offers the ability to create a diverse series of peptides, by carrying out a late stage functionalisation through the use of different linkers (Figure 6).

**Figure 7:**
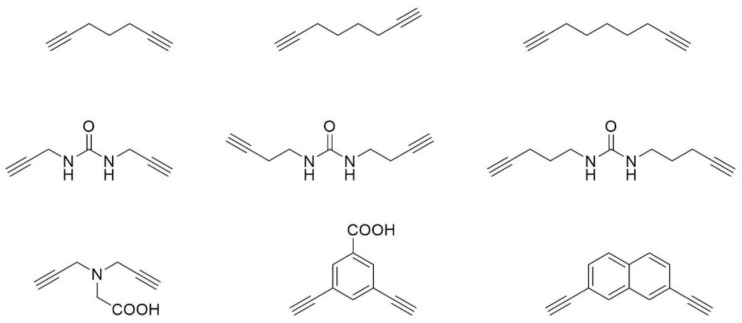
Examples of linkers that have been used for the two-component copper catalyzed azide-alkyne double-click reaction.

The Spring laboratory has pioneered a two-component peptide stapling technique based on the CuAAC reaction, which has been termed a double-click staple, where a bis-alkynyl linker is reacted with two azido containing amino acids forming two triazoles [129,144]. For this strategy, a number of different linkers (linear aliphatic and aromatic, Figure 7) have been employed through the help of molecular dynamics to target a variety of different PPIs. These include helical peptides to target the p53/MDM2 interaction [129,145,146], non-helical constrained peptides of transcription factor hepatocyte nuclear factor 1β (HNF1β) to target a key PPI in ovarian cancer [147], a macrocyclic peptide to inhibit the substrate recognition domain of tankyrase [148], and an *i,i+6* stapled helical peptide to target the genome-stability hub CTF4 [131]. A similar synthetic double-click approach has also been adopted by the Thurber group, who have reported a stabilised fluorescent GLP-1 receptor ligand exendin [133,149]. More recently, Pedersen and co-workers reported a third generation CuAAC stapling and functionalisation strategy which uses a triyne linker to give a double-clicked stapled peptide, the linker of which can then be further functionalized through another CuAAC reaction [150].

A copper free biorthogonal double strain-promoted stapling technique was also reported, which eliminates the need for the potentially toxic copper catalyst [132]. Additionally, the copper-free strategy allows for in situ stapling to generate a large library of stapled peptides directly in the assay medium in a 96-well plate.

## 5. Conclusions

Since the identification of PPIs as potential targets for therapeutics, we have witnessed their impressive journey to fame, from undruggable targets to being in the spotlight. As a result, studies into PPI stabilisers and inhibitors have increased significantly.

Although challenging, the use of small molecule PPI inhibitors has become more commonplace. Classical small molecule and peptidomimetic PPI inhibitors tend to mimic peptide sidechains to take advantage of the binding affinity of a number of hot spot residues. The cyclisation of peptides to form macrocycles has proven itself to be a valuable tool in increasing the stability of peptides in cells and in some cases also increasing the binding affinity of these peptides for their targets.

In an effort to target PPIs, stapled peptides have recently come of age, especially two stapled peptide drugs going into clinical trials. Although the gold standard for peptide stapling still remains the all-hydrocarbon staples, more and more stapling techniques are being reported to overcome some of the pitfalls of the all-hydrocarbon staple, including the need for expensive amino acids which are synthetically challenging to make and a catalyst requirement. Two-component stapling strategies are also growing in popularity due to the ease of creating a library of functionalized peptides through variation of the linker. Additionally, different approaches for stapling cysteine residues have increased in popularity, helped by the fact that there is no requirement for using non-proteinogenic amino acids. An important note is that with the wide variety of two-component cysteine stapling methodologies, a toolbox of linkers is available to use which allows researchers to specifically choose the most appropriate linkers for the job at hand, whether it be reversible [142], rigid [136], or attached to a functional handle (e.g., fluorophore, biotin etc.) [141,142,143]. Overall, in the next few decades, we foresee a series of interesting solutions to the challenges still faced by those working in the field of peptidomimetics to target PPIs.
